# A new and facile synthetic approach to substituted 2-thioxoquinazolin-4-ones by the annulation of a pyrimidine derivative

**DOI:** 10.3762/bjoc.6.120

**Published:** 2010-11-09

**Authors:** Nimalini Devi Moirangthem, Warjeet Singh Laitonjam

**Affiliations:** 1 Department of Chemistry, Manipur University, Canchipur 795 003, Manipur, India

**Keywords:** benzenoid, ethylcyanoacetate, malononitrile, pyrimidine, 2-thioxoquinazolin-4-ones

## Abstract

A new and facile synthesis of 2-thioxoquinazolin-4-ones by introducing a benzenoid system in the pyrimidine moiety by reacting ethoxymethylene derivatives of 1,3-diarylthiobarbituric acids (DTBA) with active methylene compounds, such as malononitrile and ethyl cyanoacetate, in presence of ZnCl_2_ has been developed.

## Introduction

Quinazolines and derivatives are of much interest due to their biological activities [1–2]. Additionally, quinazolines are interesting targets for new method development due to their importance in numerous therapeutic areas. Recently, antitumor [3] and anti-HIV activities [4–5] of quinazolines have been described. A large number of quinazoline derivatives, which contain the 4-oxo-2-thioxo-1,2,3,4-tetrahydropyrimidine structural moiety in their heterocyclic rings, possess a wide range of biological activities [6–8]. There are a number of synthetic methods available for the preparation of quinazolines [9]. The most common synthetic route involves the amidation of 2-aminobenzoic acid or its derivatives, i.e., 2-aminobenzonitrile, 2-aminobenzoates, and 2-arylnitrilium salts, followed by oxidative ring closure [10–13]. Other synthetic pathways include the cyclization of anthranilamides with aldehydes [14], and with ketones or acid chlorides under acidic or basic conditions [15–17]. However, most of the methods involve multistep processes and time-consuming experimental procedures, and give poor yields or use toxic reagents. Moreover, very few methods are reported for the synthesis of 2-thioxoquinazolin-4-ones, as most of the methods reported are for quinazolin-2,4(1*H*,3*H*)-diones. Recently, Saeed et al. [18] reported the base catalyzed intramolecular nucleophilic cyclization of substituted thioureas in the presence of DMF to afford 2-thioxoquinazolin-4-ones. The preparation of 2-thioxoquinazolin-4-one libraries by solid-phase synthesis has been reported [19–21].

There are two approaches for the solution-phase parallel synthesis of 2-thioxoquinazolin-4-ones [22]. The first approach is based on the reaction of methyl anthranilates with isothiocyanates in refluxing pyridine or DMF. The second approach involves briefly heating 2-(methylcarboxy)-benzeneisothiocyanates in isopropyl alcohol with a wide variety of primary aliphatic or aromatic amines and their derivatives. Thus, most of the methods for the preparation of such compounds start with the benzene ring in place followed by construction of the pyrimidine ring. We have developed a new facile and convenient synthetic approach to 2-thioxoquinazolin-4-ones by constructing the benzene ring onto an existing pyrimidine moiety.

As a part of our synthetic strategy, 1,3-diarylthiobarbituric acids (DTBA) were used as precursors for the synthesis of various fused heterocyclic compounds. In recent years, we have reported one-pot cyclizations of DTBA with hydrazine [23–24], hydroxylamine [25], guanidine [26], etc. In addition, one-pot cyclizations of DTBA-derived arylidenes have also been reported. Recently, we reported the synthesis of fused heterocycles from ethoxymethylene derivatives of DTBA [27]. In continuation of our work on the synthesis of fused heterocycles [28–29], we herein report full details of the work and studies related to the synthesis of 2-thioxoquinazolin-4-ones from the reaction of ethoxymethylene derivatives of DTBA and active methylene compounds, such as, malononitrile and ethylcyanoacetate.

## Results and Discussion

**Scheme 1:**
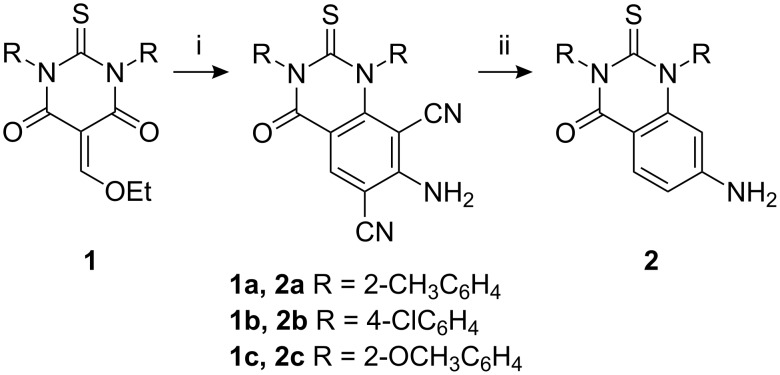
Synthesis of **2**, reagents and conditions: (i) CH_2_(CN)_2_, NH_4_OAc/AcOH, reflux, ZnCl_2_ (ii) H^+^/H_2_O.

DTBA are among the simplest synthetic intermediates and can be easily prepared in a one-pot reaction by treating 1,3-diaryl thioureas with malonic acid in the presence of acetyl chloride. DTBA undergoes condensation with ethyl orthoformate to give the condensation products, 5-ethoxymethylene-1,3-diaryl-2-thiobarbituric acids **1**. These condensation products possess three electrophilic centers and can undergo cyclocondensation with various nucleophiles to give a number of fused heterocyclic systems that contain a pyrimidine ring. Thus, treatment of **1** with malononitrile in presence of NH_4_OAc with ZnCl_2_ as catalyst in refluxing acetic acid gives the corresponding 2-thioxoquinazolin-4-ones **2** in 78–85% overall yields (Scheme 1).

During the optimization of the cyclization of ethoxymethylene derivatives of DTBA with malononitrile, the choice of the base proved to be an important parameter. The use of NEt_3_ (TEA) or piperidine (in DCM or ethanol) resulted in the formation of complex mixtures (Table 1, entries 1–4). Screening at different temperatures demonstrates that some of the catalysts failed to react at room temperature (rt) and also even after heating under reflux (Table 1, entries 5–8). The reaction failed with both ZnCl_2_ and FeCl_3_ in MeOH solution at rt. Similarly, no reaction was observed with NaOCH_3_ and MeOH at rt. On the other hand, with NaOCH_3_ as base in MeOH and ZnCl_2_ as catalyst at rt, a relatively low yield of **2a** was obtained (Table 1, entry 9). However, when this reaction was repeated in refluxing solvent the yield was increased.

**Table 1:** Effect of base and solvent on the yield for the synthesis of **2a**.

Entry	Conditions	Yield^c^ (%)

1	NEt_3_ in DCM	no product
2	NEt_3_ in EtOH	no product
3	piperidine in DCM	no product
4	piperidine in EtOH	no product
5	ZnCl_2_ in MeOH^a,b^	no product
6	FeCl_3_ in MeOH^a,b^	no product
7	AlCl_3_ in MeOH ^a,b^	no product
8	NaOCH_3_ in MeOH^a^	no product
9	NaOCH_3_ in MeOH^b^	24
10	NaOCH_3_ in MeOH, ZnCl_2_^b^	35
11	NH_4_OAc in AcOH^b^	67
12	NH_4_OAc in AcOH^b^	85

^a^Reactions were carried out at room temperature. ^b^Reactions were carried out at reflux. ^c^Isolated yield.

In contrast, the use of NH_4_OAc in refluxing acetic acid resulted in a clean cyclization to give the desired product. Dehydration and decarboxylation induced by the higher temperature and the acid produces the required quinazoline. To obtain the optimal conditions, a variety of catalysts were also investigated to detect the catalytic activities of different metal ions and acetate in the production of **2a** (Table 2). It was found that NH_4_OAc/AcOH in ZnCl_2_ was the most effective (Table 2, entries 1 and 7–13); CuCl_2_ and HgCl_2_ also promoted the reactions, but the yields were poor, 22% and 12%, respectively (Table 2, entries 2 and 3). Other catalysts, including FeCl_3_, AlCl_3_ etc. failed to afford any **2a** (Table 2, entries 4–6). We further found that the best yield of **2a** was obtained when 5 equiv of ZnCl_2_ was used (Table 2, entry 13). The excessive amount of ZnCl_2_ for the annulation is probably due to the chelating effect of zinc ion. Thus, the NH_4_OAc/AcOH combination in ZnCl_2_ was found to be the best and gave the highest yield of **2b** (85%) after refluxing for 6 h.

**Table 2:** Effect of catalysts in the yield for synthesis of **2a**^a^.

Entry	Conditions	Yield^b^ (%)

1	ZnCl_2_ (1 equiv)^a^	30
2	CuCl_2_ (1 equiv)^a^	22
3	HgCl_2_ (1 equiv)^a^	12
4	FeCl_3_ (1 equiv)^a^	0
5	AlCl_3_ (1 equiv)^a^	0
6	SnCl_2_ (1 equiv)^a^	0
7	ZnCl_2_ (0.5 equiv)^a^	18
8	ZnCl_2_ (2 equiv)^a^	45
9	ZnCl_2_ (5 equiv, 2 h)^a^	62
10	ZnCl_2_ (5 equiv, rt, 4 h)	28
11	ZnCl_2_ (5 equiv, rt, 6 h)	38
12	ZnCl_2_ (5 equiv, 4 h)^a^	74
13	ZnCl_2_ (5 equiv, 6 h)^a^	85

^a^Reactions were carried out with NH_4_OAc and AcOH at reflux. ^b^Isolated yield.

**Scheme 2:**
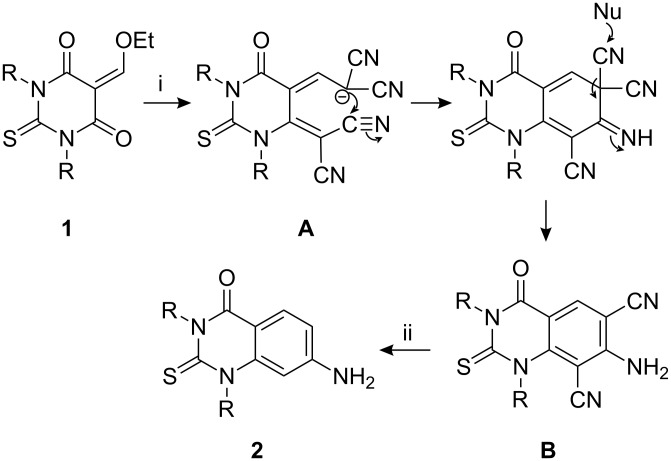
Synthesis of **2**, reagents and conditions: (i) CH_2_(CN)_2_, NH_4_OAc/AcOH, reflux, ZnCl_2_ (ii) H^+^/H_2_O.

After optimizing the conditions, various DTBAs were used to react with malononitrile and the results are listed in Table 3. On the basis of the above noted results, a possible reaction mechanism is shown in Scheme 2. The reaction of the 5-ethoxymethylene-1,3-diaryl-2-thiobarbituric acids with malononitrile gave intermediate **A**, which undergoes intramolecular cyclization to form the intermediate **B**, and then acid hydrolysis of **B** afforded **2**. Further evidence is that the reaction of 5-ethoxymethylene-1,3-diaryl-2-thiobarbituric acids with malononitrile under the standard conditions. This reaction only gave quinazolines and no other products were detected. In addition, this proposed mechanism was also confirmed from the literature [30–31].

**Table 3:** Synthesis of 7-amino-2,3-dihydro-2-thioxo-1,3-diarylquinazolin-4(1*H*)-ones^a^.

Product	R	Yield (%)^b^

**2a**	2-CH_3_C_6_H_4_	83
**2b**	4-ClC_6_H_4_	85
**2c**	2-OCH_3_C_6_H_4_	78

^a^Reactions were carried out with NH_4_OAc and AcOH at reflux. ^b^Isolated yield.

**Scheme 3:**
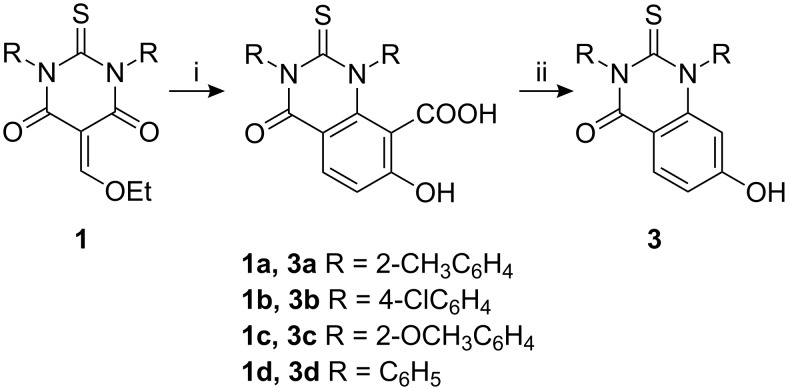
Synthesis of **3**, reagents and conditions: (i) NC-CH_2_-CO_2_Et, NH_4_OAc/AcOH, reflux, ZnCl_2_ (ii) H_3_O^+^.

The reaction of 5-ethoxymethylene-1,3-diaryl-2-thiobarbituric acids **1** with ethylcyanoacetate in presence of ammonium acetate and acetic acid with ZnCl_2_ as a catalyst afforded 7-hydroxy-2,3-dihydro-2-thioxo-1,3-diarylquinazolin-4(1*H*)-ones **3** in 76–87% overall yields (Scheme 3) [32–33] and these results are listed in Table 4.

**Table 4:** Synthesis of 7-hydroxy-2,3-dihydro-2-thioxo-1,3-diarylquinazolin-4(1*H*)-ones^a^.

Product	R	Yield (%)^b^

**3a**	2-CH_3_C_6_H_4_	82
**3b**	4-ClC_6_H_4_	87
**3c**	2-OCH_3_C_6_H_4_	80
**3d**	C_6_H_5_	76

^a^Reactions were carried out with NH_4_OAc and AcOH at reflux. ^b^Isolated yield.

## Conclusion

The cyclocondensation of ethoxymethylene thiobarbituric acids with active methylene compounds under the above noted catalytic system, resulted in a new method for the formation of quinazoline derivatives. Thus, the reaction of 5-ethoxymethylenepyrimidine-4,6-diones **1** with malononitrile and ethyl cyanoacetate gave 7-amino-2,3-dihydro-2-thioxo-1,3-diarylquinazolin-4(1*H*)-ones **2** and 7-hydroxy-2,3-dihydro-2-thioxo-1,3-diarylquinozolin-4(1*H*)-ones **3**, respectively. This new procedure avoids the use of toxic reagents which are traditionally used for the preparation of quinazolines.

## Supporting Information

File 1 Experimental part.

File 2 IR and NMR spectra.
Supporting Information feature copies of IR and ^1^H NMR spectra of 7-amino-2,3-dihydro-2-thioxo-1,3-di(2-methoxyphenyl)quinazolin-4(1*H*)-one (**2c**) and ^1^H and ^13^C NMR spectra of 7-hydroxy-2,3-dihydro-2-thioxo-1,3-di(2-methylphenyl)quinazolin-4(1*H*)-one (**3a).**
