# Supplementary material for: A new and facile synthetic approach to substituted 2-thioxoquinazolin-4-ones by the annulation of a pyrimidine derivative
Source: Beilstein J Org Chem. 2010 Nov 9;6:1056–60. doi: 10.3762/bjoc.6.120 (PMC2981811; doi:10.3762/bjoc.6.120)
Supplement: File 1 — Experimental part. [file Beilstein_J_Org_Chem-06-1056-s001.pdf]

Supporting Information

for

A new and facile synthetic approach to substituted 2-thioxoquinazolin-4-ones through annulation of aromatic rings onto pyrimidine derivatives

Nimalini D. Moirangthem and Warjeet S. Laitonjam*

Address: Department of Chemistry, Manipur University, Canchipur 795 003, Manipur, India

Email: Warjeet S. Laitonjam - warjeet@yahoo.com

Nimalini Devi Moirangthem - nima_moirangthem@rediffmail.com

* Corresponding author

Experimental part

General procedure for the synthesis of 7-amino-2,3-dihydro-2-thioxo-1,3-di(2-methylphenyl)quinazolin-4(1*H*)-one, 2a:

A mixture of 1,3-di(2-methylphenyl)-1,3-dihydro-2-thioxo-5-ethoxymethylene-pyrimidine-4,6-dione (**1a**) (0.4 g, 0.001 mol) and malononitrile (0.3 g, 0.004 mol) were heated under reflux in the presence of NH_4OAc (1 equiv), AcOH (1 equiv) and $ZnCl_2$ (6 equiv) at 120 °C for 6 h (monitored by TLC). The reaction mixture was diluted with $CHCl_3$ and water. The organic layer

was separated and dried over anhydrous Na_2SO_4. The solvent was concentrated in vacuo and then purified by column chromatography with PE and EA as eluent. Compound **2a** was obtained as an amorphous yellow solid in 83% yield, mp 180–182 °C; IR/KBr/cm^{-1}: 3346, 3225, 2951, 1682, 1647, 1510, 1416, 1331, 1273, 1157, 1107, 881, 808; ^{1}H NMR (400 MHz, $CDCl_3$): δ 9.38 (1H, d, *J* = 7.6 Hz), 8.18 (1H, d, *J* = 7.6 Hz), 7.32 (1H, m), 7.25–7.32 (5H, m, Ar), 7.09-7.14 (3H, m, Ar), 2.40 (3H, s, CH_3), 2.37 (3H, s, CH_3); ^{13}C NMR (100 MHz, $CDCl_3$): δ 181.14, 163.49, 162.46, 160.79, 138.76, 138.75, 137.14, 136.38, 130.27, 128.41, 128.37, 93.42, 60.46,21.36, 21.35; Mass(EI): *m/z* 374 [M+1].

Compounds **2b** and **2c** were prepared similarly.

7-Amino-2,3-dihydro-2-thioxo-1,3-di(4-chlorophenyl)quinazolin-4(1*H*)-one (2b): Yield: 85%; mp 220–221 °C; IR/KBr/cm^{-1}: 3050, 2980, 1705, 1622, 1562, 1319, 1269, 1092, 810; ^{1}H NMR (400 MHz, $CDCl_3$): δ 12.19 (1H, d, *J* = 7.6 Hz), 8.79 (1H, d, *J* = 7.6 Hz), 7.46 (1H, m), 7.19–7.34 (3H, m, Ar), 6.93–7.12 (5H, m, Ar), 3.86 (3H, s, OCH_3), 3.83 (3H, s, OCH_3); ^{13}C NMR (100 MHz, $CDCl_3$): δ 180.85,163.58, 162.58, 161.06, 130.53, 130.30, 130.06, 130.11, 128.88, 128.66, 128.19, 121.44, 121.31, 112.96, 112.61, 93.57, 56.52, 56.34; Mass(EI) : *m/z* 415 [M+1].

7-Amino-2,3-dihydro-2-thioxo-1,3-di(2-methoxyphenyl)quinazolin-4(1*H*)-one (2c): Yield: 78%; mp 188–190 °C; IR/KBr/cm^{-1}: 3072, 2941, 2839, 1686, 1651, 1452, 1327, 1021, 750; ^{1}H NMR (400 MHz, $CDCl_3$): δ 12.19 (1H, d, *J* = 7.6 Hz), 8.79 (1H, d, *J* = 7.6 Hz), 7.46 (1H, m), 7.19–7.34 (3H, m, Ar), 6.93–7.12 (5H, m, Ar), 3.86 (3H, s, OCH_3), 3.83 (3H, s, OCH_3); ^{13}C NMR (100 MHz, $CDCl_3$): δ 180.85,163.58, 162.58, 161.06, 130.53, 130.30, 130.06, 130.11, 128.88, 128.66, 128.19, 121.44, 121.31, 112.96, 112.61, 93.57, 56.52, 56.34; Mass(EI): *m/z* 405.

General procedure for the synthesis of 7-hydroxy-2,3-dihydro-2-thioxo-1,3-di(2-methylphenyl)quinazolin-4(1*H*)-one (3a):

1,3-Di(2-methylphenyl)-1,3-dihydro-2-thioxo-5-ethoxymethylene-pyrimidine-4,6-dione (**1a**) (0.4 g, 0.001 mol) and ethylcyanoacetate (0.3 mL, 2.6 mol) were heated under reflux in the presence of NH_4OAc, AcOH and $ZnCl_2$ (6 equiv) at 120 °C for 6 h in an oil bath. The reaction mixture was treated as above. The organic layer was dried over anhydrous Na_2SO_4 and the solvent was concentrated in vacuo and then purified by column chromatography with PE and EA as eluent. The product **3a** was obtained as an amorphous yellow solid in 82% yield, mp 170–171 °C; IR/KBr/cm^{-1}: 3379, 3034, 1686, 1645, 1612, 1456, 1329, 1265, 810; ^1H NMR (400 MHz, $CDCl_3$): δ 12.23 (1H, d, *J* = 7.6 Hz), 8.74 (1H, d, *J* = 7.6 Hz), 7.35 (1H, m), 7.21–7.31 (5H, m, Ar), 7.03–7.18 (3H, m, Ar), 2.48 (3H, s, CH_3), 2.41 (3H, s, CH_3); ^{13}C NMR (100 MHz, $CDCl_3$): δ 180.94, 164.03, 161.62, 153.02, 138.81, 138.48, 137.45, 137.15, 136.38, 135.22, 130.75, 130.32, 130.20, 128.37, 128.31, 117.99, 94.44, 21.39, 20.98; Mass(EI): *m/z* 375 [M+1].

Compounds **3b**, **3c** and **3d** were prepared similarly.

7-Hydroxy-2,3-dihydro-2-thioxo-1,3-di(4-chlorophenyl)quinazolin-4(1*H*)-one (3b): Yield: 87%; mp 210–212 °C; IR/KBr/cm^{-1}: 3030, 1708, 1571, 1440, 1319, 1260, 780; ^1H NMR (400 MHz, $CDCl_3$): δ 12.19 (1H, d, *J* = 7.6 Hz), 8.72 (1H, d, *J* = 7.6 Hz), 7.53 (1H, m), 7.42–7.51 (4H, m, Ar), 7.17–7.28 (4H, m, Ar); ^{13}C NMR (100 MHz, $CDCl_3$): δ 180.25, 163.68, 161.04, 153.24, 137.82, 137.06, 136.02, 135.01, 134.70, 133.10, 130.48, 130.13, 130.07, 129.83, 119.37, 94.78; Mass(EI): *m/z* 416 [M+1].

7-Hydroxy-2,3-dihydro-2-thioxo-1,3-di(2-methoxyphenyl)quinazolin-4(1*H*)-one (3c):

Yield: 80%; mp 160–162 °C; IR/KBr/cm^{-1}: 3576, 3069, 2941, 1686, 1649, 1450, 1331, 1281, 1018, 750; ^{1}H NMR (400 MHz, $CDCl_3$): δ 12.18 (1H, d, *J* = 7.6 Hz), 8.79 (1H, d, *J* = 7.6 Hz), 7.46 (1H, m), 7.39–7.44 (4H, m, Ar), 7.05–7.33 (4H, m, Ar), 3.87 (3H, s, OCH_3), 3.82 (3H, s, OCH_3); ^{13}C NMR (100 MHz, $CDCl_3$): δ 180.61, 163.23, 161.62, 155.04, 154.84, 152.03, 56.29, 56.12; Mass(EI): *m/z* 406 [M+1].

7-Hydroxy-2,3-dihydro-2-thioxo-1,3-diphenylquinazolin-4(1*H*)-one (3d):

Yield: 76%; mp 192–194 °C; IR/KBr/cm^{-1}: 3512, 3035, 2911, 1678, 1645, 725; ^{1}H NMR (400 MHz, $CDCl_3$): δ 12.12 (1H, d, *J* = 7.6 Hz), 8.69 (1H, d, *J* = 7.6 Hz), 7.52 (1H, m), 7.28–7.46 (4H, m, Ar), 7.15–7.24 (4H, m, Ar); ^{13}C NMR (100 MHz, $CDCl_3$): δ 179.88, 163.59, 161.12, 152.82, 137.66, 137.12, 135.06, 92.71; Mass(EI): *m/z* 347 [M+1].

Table 1: Characteristic data of quinazolines (**2a–c** and **3a–d**).

Compound	mp (°C)	Yield (%)	Molecular formula (mol. Mass)
**2a**	180–182	83	$C_{22}H_{19}N_3OS$ (373)
**2b**	220–221	85	$C_{20}H_{13}N_3Cl_2OS$ (414)
**2c**	188–190	78	$C_{22}H_{19}N_3O_3S$ (405)
**3a**	170–171	82	$C_{22}H_{18}N_2O_2S$ (374)
**3b**	210–212	87	$C_{20}H_{12}N_2Cl_2O_2S$ (415)
**3c**	160–162	80	$C_{22}H_{18}N_2O_4S$ (406)
**3d**	192–194	76	$C_{20}H_{14}N_2O_2S$ (346)
