# Supplementary material for: A new and facile synthetic approach to substituted 2-thioxoquinazolin-4-ones by the annulation of a pyrimidine derivative
Source: Beilstein J Org Chem. 2010 Nov 9;6:1056–60. doi: 10.3762/bjoc.6.120 (PMC2981811; doi:10.3762/bjoc.6.120)
Supplement: File 2 — IR and NMR spectra. Supporting Information feature copies of IR and 1H NMR spectra of 7-amino-2,3-dihydro-2-thioxo-1,3-di(2-methoxyphenyl)quinazolin-4(1H)-one (2c) and 1H and 13C NMR spectra of 7-hydroxy-2,3-dihydro-2-thioxo-1,3-di(2-methylphenyl)quinazolin-4(1H)-one (3a). [file Beilstein_J_Org_Chem-06-1056-s002.pdf]

Supporting Information

for

A new and facile synthetic approach to substituted 2-thioxoquinazolin-4-ones through annulation of aromatic rings onto pyrimidine derivatives

Nimalini D. Moirangthem and Warjeet S. Laitonjam*

Address: Department of Chemistry, Manipur University, Canchipur 795 003, Manipur, India

Email: Warjeet S. Laitonjam - warjeet@yahoo.com

Nimalini Devi Moirangthem - nima_moirangthem@rediffmail.com

* Corresponding author

IR and NMR spectra

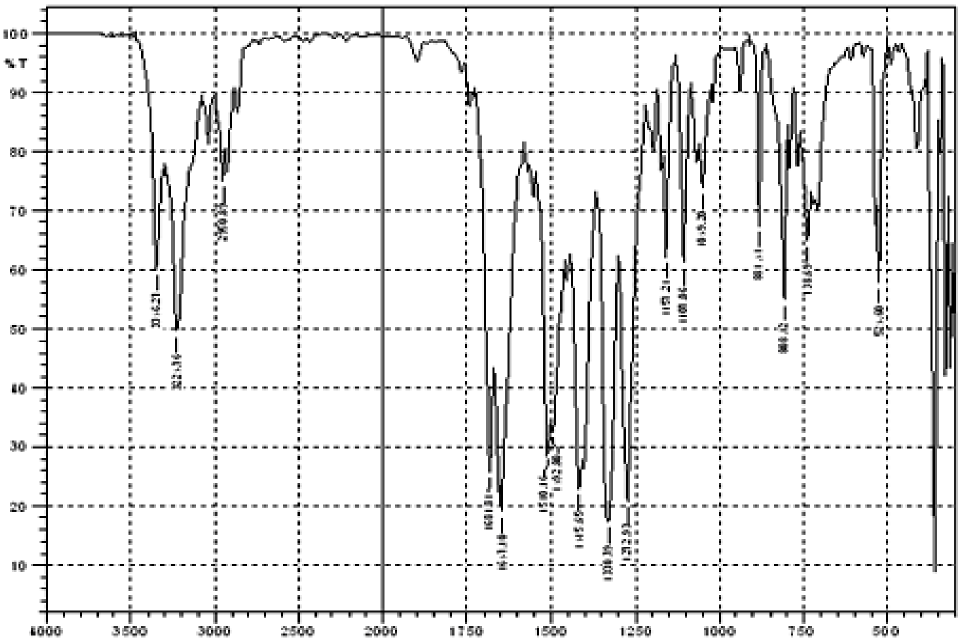

Figure 1: IR spectrum of compound **2c**.

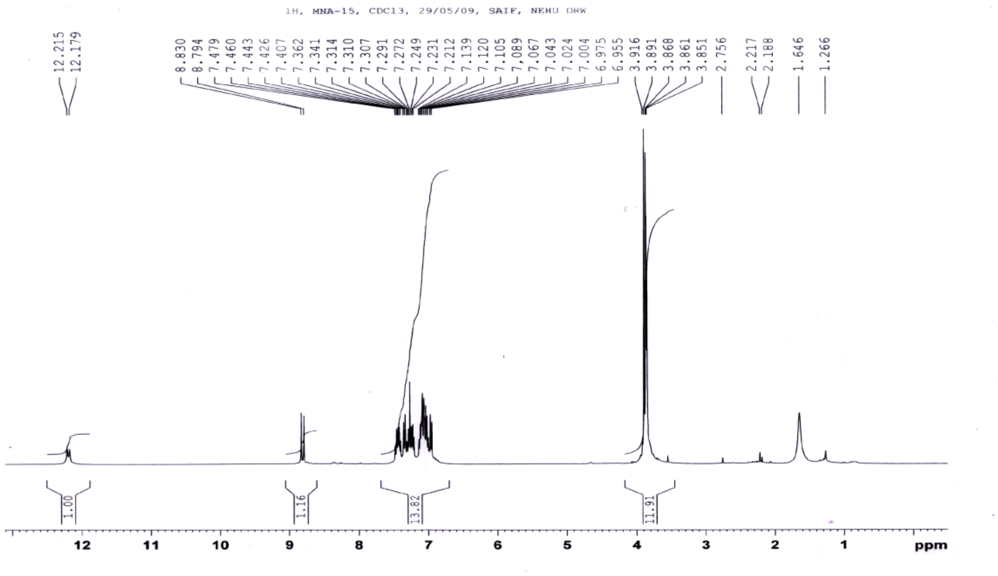

Figure 2: ^{1}H NMR spectrum of **2c**.

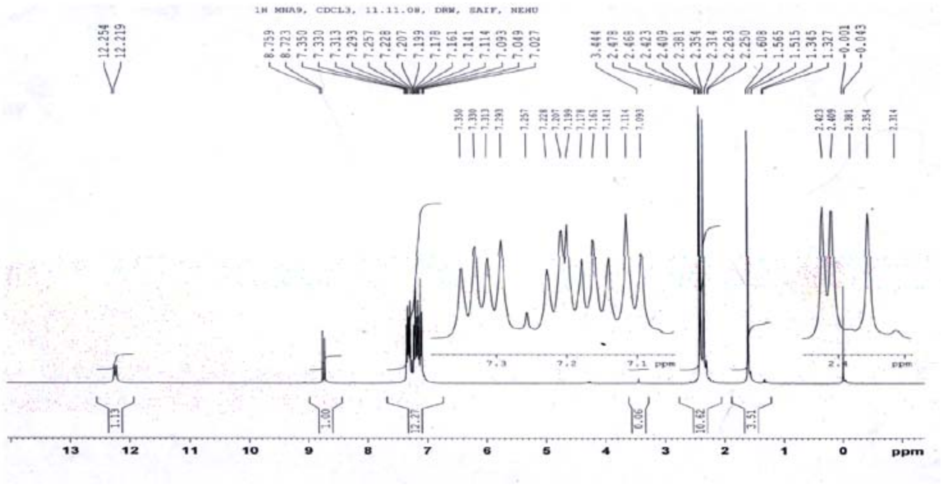

Figure 3: ^{1}H NMR spectrum of **3a**.

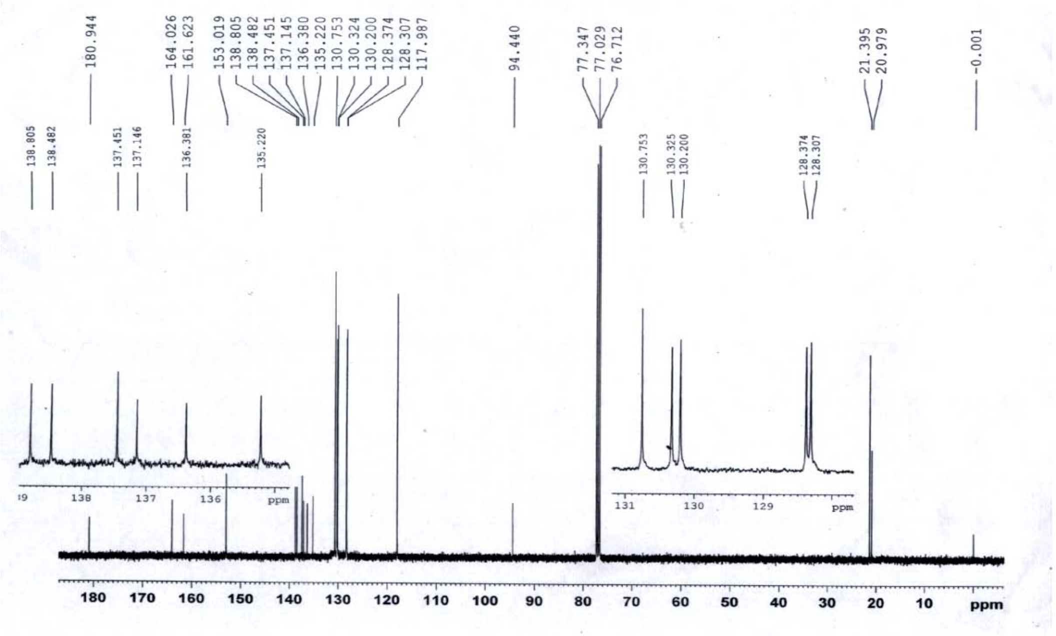

Figure 4: ^{13}C NMR spectrum of **3a**.
